# A systems radiation biology approach to unravel the role of chronic low-dose-rate gamma-irradiation in inducing premature senescence in endothelial cells

**DOI:** 10.1371/journal.pone.0265281

**Published:** 2022-03-14

**Authors:** Gabriele Babini, Giorgio Baiocco, Sofia Barbieri, Jacopo Morini, Traimate Sangsuwan, Siamak Haghdoost, Ramesh Yentrapalli, Omid Azimzadeh, Charlotte Rombouts, An Aerts, Roel Quintens, Teni Ebrahimian, Mohammed Abderrafi Benotmane, Raghda Ramadan, Sarah Baatout, Soile Tapio, Mats Harms-Ringdahl, Andrea Ottolenghi

**Affiliations:** 1 Physics Department, University of Pavia, Pavia, Italy; 2 Faculty of Medicine, Department of Cellular Physiology and Metabolism, University of Geneva, Geneva, Switzerland; 3 Department of Molecular Bioscience, Centre for Radiation Protection Research, Wenner-Gren Institute, Stockholm University, Stockholm, Sweden; 4 ARIA Laboratory, University of Caen Normandy, CIMAP-GANIL, 14076, Caen, France; 5 Institute of Radiation Biology, Helmholtz Zentrum Muenchen—German Research Centre for Environmental Health, Neuherberg, Germany; 6 Section Radiation Biology, Federal Office for Radiation Protection, Munich, Germany; 7 Radiobiology Unit, Belgian Nuclear Research Centre, SCK CEN, Boeretang, Belgium; 8 Department of Molecular Biotechnology, Ghent University, Ghent, Belgium; 9 Laboratoire de Radiobiologie et RadioToxicologie expérimentale, Service de recherche des effets biologiques et sanitaires des rayonnements ionisants, Pôle santé, F-92262, Fontenay-aux-Roses, France; Northwestern University Feinberg School of Medicine, UNITED STATES

## Abstract

**Purpose:**

The aim of this study was to explore the effects of chronic low-dose-rate gamma-radiation at a multi-scale level. The specific objective was to obtain an overall view of the endothelial cell response, by integrating previously published data on different cellular endpoints and highlighting possible different mechanisms underpinning radiation-induced senescence.

**Materials and methods:**

Different datasets were collected regarding experiments on human umbilical vein endothelial cells (HUVECs) which were chronically exposed to low dose rates (0, 1.4, 2.1 and 4.1 mGy/h) of gamma-rays until cell replication was arrested. Such exposed cells were analyzed for different complementary endpoints at distinct time points (up to several weeks), investigating cellular functions such as proliferation, senescence and angiogenic properties, as well as using transcriptomics and proteomics profiling. A mathematical model was proposed to describe proliferation and senescence.

**Results:**

Simultaneous ceasing of cell proliferation and senescence onset as a function of time were well reproduced by the logistic growth curve, conveying shared equilibria between the two endpoints. The combination of all the different endpoints investigated highlighted a dose-dependence for prematurely induced senescence. However, the underpinning molecular mechanisms appeared to be dissimilar for the different dose rates, thus suggesting a more complex scenario.

**Conclusions:**

This study was conducted integrating different datasets, focusing on their temporal dynamics, and using a systems biology approach. Results of our analysis highlight that different dose rates have different effects in inducing premature senescence, and that the total cumulative absorbed dose also plays an important role in accelerating endothelial cell senescence.

## Introduction

Cardiovascular disease (CVD) is one of the major causes of morbidity and mortality in Western countries, including a wide range of pathologies related to the heart, blood vessels and brain vasculature [1]. Populations exposed to ionizing radiation, either for occupational, therapeutic and diagnostic or environmental reasons, show increased risk of cardiovascular disorders [2–4].

This knowledge was obtained through several epidemiological studies conducted over the last decades, among which studies on the Japanese atomic bomb survivors, including the Life Span Study cohort and the Adult Health Study cohort, are the best known [5–7]. Although there are still some uncertainties on the dose reconstruction, these epidemiological analyses highlight an increased risk of stroke and heart disease, leading to about one-third of the radiation-associated excess deaths [8,9]. Data regarding CVD risk at protracted exposures have mainly been obtained from occupational cohorts, such as Chernobyl liquidators [10], National Dose Registry of Canada and British Nuclear Fuels plc [11–13] and Mayak nuclear facility workers [14]. All of these studies showed an increasing trend in circulatory disease morbidity and mortality, with injuries to the cardiovascular system, in particular, showing heart failure [15,16].

Another important area of evidence of radiation-induced CVD comes from the long-term follow-up of radiotherapy patients, such as breast cancer and Hodgkin lymphoma patients. One of the most striking evidence of radiation-induced CVD was observed by comparing patients with left-sided breast cancers to those with right-sided ones, showing a dose-dependent increase of heart morbidity and mortality in the former group [17–19]. This could be explained by the higher cumulative dose received by the heart from radiotherapy in left-sided breast cancer patients, which is estimated to be approximately 6.6 Gy, compared to 2.9 Gy in women with right-sided breast cancer [20]. With an increasing number of cancer survivors, the number of patients showing long-term radiotherapy-induced CVD is also increasing, demanding for a more careful treatment planning and countermeasures to prevent such long-term side effects [21].

Among others, the vascular endothelium, the inner layer of blood vessels separating tissues from circulating blood, is a critical target for radiation-induced CVDs. Endothelial cells have several functions, such as the maintenance of vascular homeostasis, vasodilatation and -constriction of the arterial walls, and control of blood cell trafficking [22]. Any dysfunction to the endothelial layer generates a loss of important properties for safeguarding the normal vascular function, possibly leading to the onset and progression of CVD. Among the possible causes of endothelial dysfunctions, reactive oxygen species (ROS) are known to be increasingly produced by endothelial cells during the entire human aging process [23]. This might lead to premature endothelial aging, which may impair the normal blood flow and thereby contribute to cellular senescence and inflammation, which accelerate the formation of atherosclerotic plaques and eventually lead to myocardial infarction and stroke [24,25].

The scenario of radiation-induced diseases is even more debated when analysing and interpreting the damaging impact of low doses (below 100 mGy) and low dose rates. Several studies using *in vitro* and *in vivo* models showed either potential benefits of chronic exposures [26–28], or adverse effects [29]. In particular, for the latter case, the observed effects included the induction of translocations, micronucleus formations [30] and the induction of ROS [31] that might eventually lead to radiation-induced genotoxic effects. These data support the hypothesis that low-dose-rate radiation may eventually induce both cancer and non-cancer diseases [32–34].

Within this general context, a specific task was included in the European project EURATOM FP7 NoE "DoReMi" to shed light on the effects of protracted gamma-ray exposures of Human umbilical vein endothelial cells (HUVECs) at the proteomics and transcriptomic levels and by investigating some end points such as proliferation, vascular network formation and senescence. The results of the consortium showed a dose-rate dependent decrease in cell proliferation and concomitant increase in senescence-stained HUVECs. Further analysis at the proteome level showed clear signatures of proteomic changes that were involved mainly in the PI3K/Akt/mTOR pathway and oxidative stress [35,36], while gene expression profiling demonstrate an early stress response characterized by the expression of inflammation-related genes that were hypothesized to activate the onset of the radiation-induced premature senescence via the IGFBP5 signaling pathway [37].

In this study we used system biology approach, a valuable tool for understanding and even predicting the details of complex biological systems, providing a more holistic molecular perspective and deeper understanding of studied biological systems by integrating multiple layers of quantitative molecular events with well-designed mathematical models [38]. The aim of the present study was to collect and integrate such previously obtained experimental results with a systems biology approach, and to provide a phenomenological interpretation of the observed premature induction of cellular senescence. Particularly, the attention was directed towards complementary endpoints and the investigation of dose-rate-dependent phenomena. Using a systems biology approach, the integration of different omics studies and their comparison at similar doses (different times of exposure to different dose rates) provided a deeper insight on the underpinning mechanisms of radiation-induced cellular senescence, not only pointing towards a total cumulative dose-dependence but also suggesting an important role of the dose rate at which the total dose was delivered.

## Materials and methods

### Cell culture, irradiation and calculation of population doubling

The datasets discussed within this work were obtained from experiments performed on HUVECs (Invitrogen, Paisley, UK) as described in details in previous publications [35,36]. HUVECs were cultured in Media 200 (Invitrogen) with low serum growth supplements (2% FBS) were passaged every seven days (5000 cells/cm^2^) and culture medium was changed every two days. Irradiation was carried out continuously using a customized cell culture incubator with a ^137^Cs-gamma source, available at Stockholm University. Dose rates were 1.4, 2.4 and 4.1 mGy/h. Control cells were grown in an identical incubator but without exposure to ionizing radiation. At each passage, growth rate kinetics were established counting the cells prior to sub-culturing and calculating the Population Doubling (PD) as:

$$PD\left(t\right)={\text{log}}_{2}\left(\frac{N\left(t\right)}{N_{0}}\right)$$ (1)

*N* represent the number of complete sets of cells, where ***N***_***0***_ is the initial number of cells seeded and ***N(t)*** refers to the number of cells counted at week *"****t****"*. In this work, the ratio ***N(t)/ N***_***0***_ entering in the definition of the population doubling is used to quantify cell growth. Three independent experiments were performed as biological replicates.

To calculate the "total cumulative dose", exposure time was multiplied by the dose rate (e.g. the total cumulative dose for 1.4 mGy/h at 1 week = 1.4 × 24 × 7 = 0.24 Gy). Thus, the total cumulative doses at 1, 3, 6 and 10 weeks for 1.4 mGy/h were 0.24, 0.71, 1.41 and 2.3 Gy, respectively, while for 4.1 mGy/h the total cumulative doses were 0.69, 2.1, 4.1 and 6.9 Gy, respectively [39].

### Assay of Senescence-Associated-β-galactosidase (SA-β-gal)

The study on radiation-induced senescence was conducted on data obtained from SA-β-gal measurements determined by a histochemical staining kit, according to the manufacturer’s instructions (Sigma-Aldrich, Sweden). The percentage of SA-β-gal stained cells was scored at different time points: weeks = 1, 3, 6, 10, 12, 15 or until cells stopped growing [35,36].

### Vascular network formation

The analysed results were obtained from cells which were first frozen at different passages, after receiving different total cumulative radiation doses, and then thawed and allowed to grow for three days either in normal culture medium or on Matrigel. In the former case, the culture medium was collected and IL-6 was measured, while in the latter case the capacity to form vascular networks on Matrigel was assessed as described in detail in Ebrahimian et al. [39].

### Theoretical analysis

To study radiation-induced effects of chronic exposures on the population growth and senescence of HUVECs we adopted an analytical approach.

Given the initial seed concentration and the time allowed for growing, the most appropriate growth curve is the logistic growth function. The reason for this choice lies in the fixed maximum number of cells that can fit on a monolayer of culture flasks and in the limited amount of nutrients in the culture medium. For this reason, the growth rate becomes smaller when the population approaches the maximum available size. This can be mathematically described as follows [McKendrick, et al. 1912; Gleb 2012]:

$$dN\left(t\right)=r*N*\left(1-N/K\right)dt$$ (2)

where ***dN*** is the increment in cell number in time interval ***dt***, ***r*** and ***1/K*** are referred to the growth rate parameter and the inverse of the carrying capacity, respectively.

The solution of the differential equation can be given as follows:

$$\frac{N\left(t\right)}{N_{0}}=\frac{K}{1+A*e^{-rt}}$$ (3)

where:

$$A=\frac{K-N_{0}}{N_{0}}$$ (4)

The free parameters ***K***, (hence ***A***) and ***r*** are obtained from the fit of experimental data.

The same model is adopted to describe the time dependence of the percentage ***S(t)*** of senescent (positive to β-galactosidase staining) cells:

$$S\left(t\right)=\frac{100}{1+B*e^{-pt}}$$ (5)

Where the free parameters are only **B** and ***p***, since, by definition, the maximum value for ***S(t)*** for ***t→∞*** is 100%.

For all fits to experimental data and data visualizations we used Gnuplot Version 5.0 open source software (http://www.gnuplot.info). The *"fit"* command used in Gnuplot can fit the user-defined functions (Eqs (3) and (5)) to a set of data points (x, y, y-standard-deviation), using an implementation of the nonlinear least-squares (NLLS) Marquardt-Levenberg algorithm [40,41].

Uncertainties on fit parameters (commonly called standard deviations) are given as output of the fit routine and calculated as detailed in (http://gnuplot.sourceforge.net/docs_5.0/gnuplot.pdf).

### Analysis of transcriptomics and proteomics datasets

Within DoReMi Task 7.3 project, both RNA and proteins were extracted from cell lysates and underwent high-throughput screening to explore possible changes in the transcriptome and proteome at different times (1 week, 3, 6 and 10 weeks) and at the different dose rates used for chronic exposure (An overview of the available dose rates and time points of the transcriptomic and proteomic data is available in S1 Table). A detailed description of methods and results for the microarray data analysis and for the proteome profiling is given in the works by (Rombouts et al. 2014 [37], raw data available at ArrayExpress E-MTAB-6521) and (Yentrapalli, Azimzadeh, Sriharshan, et al. 2013 [35], raw data available at https://www.storedb.org/store_v3/study.jsp?studyId=1105; Yentrapalli, Azimzadeh, Barjaktarovic, et al. 2013 [36], raw data available at https://www.storedb.org/store_v3/study.jsp?studyId=1104, respectively).

In this work, we present results based on the integration of these two datasets (transcriptomics and proteomics) as described previously by Haider et al, 2013 [42]. For the integration, we unified the transcriptomic and proteomic levels for each of the conditions and time points available. The merged datasets of significant proteins/mRNA were then analyzed through the “Reactome FI” Cytoscape plugin [43].

Pathway enrichment was performed via the "Gene set" analysis in which the protein/mRNA list was loaded as a file containing one entry per line. This method produces a network reconstruction, enriched with linker genes (*i*.*e*. another gene connected to a gene of the input list), and provides information about the statistical significance (p-values and False Discovery Rate "FDR") of the pathways to which the entries are belonging. In particular, statistical significance (p-value) was calculated using Fisher’s exact test while FDR calculation is based on the Benjamini–Hochberg method for multiple comparisons [44]. The obtained pathways were then compared among the different dose-rate conditions, time points, and total cumulative doses (whenever possible).

## Results

### Cell population doublings

**Fig 1:**
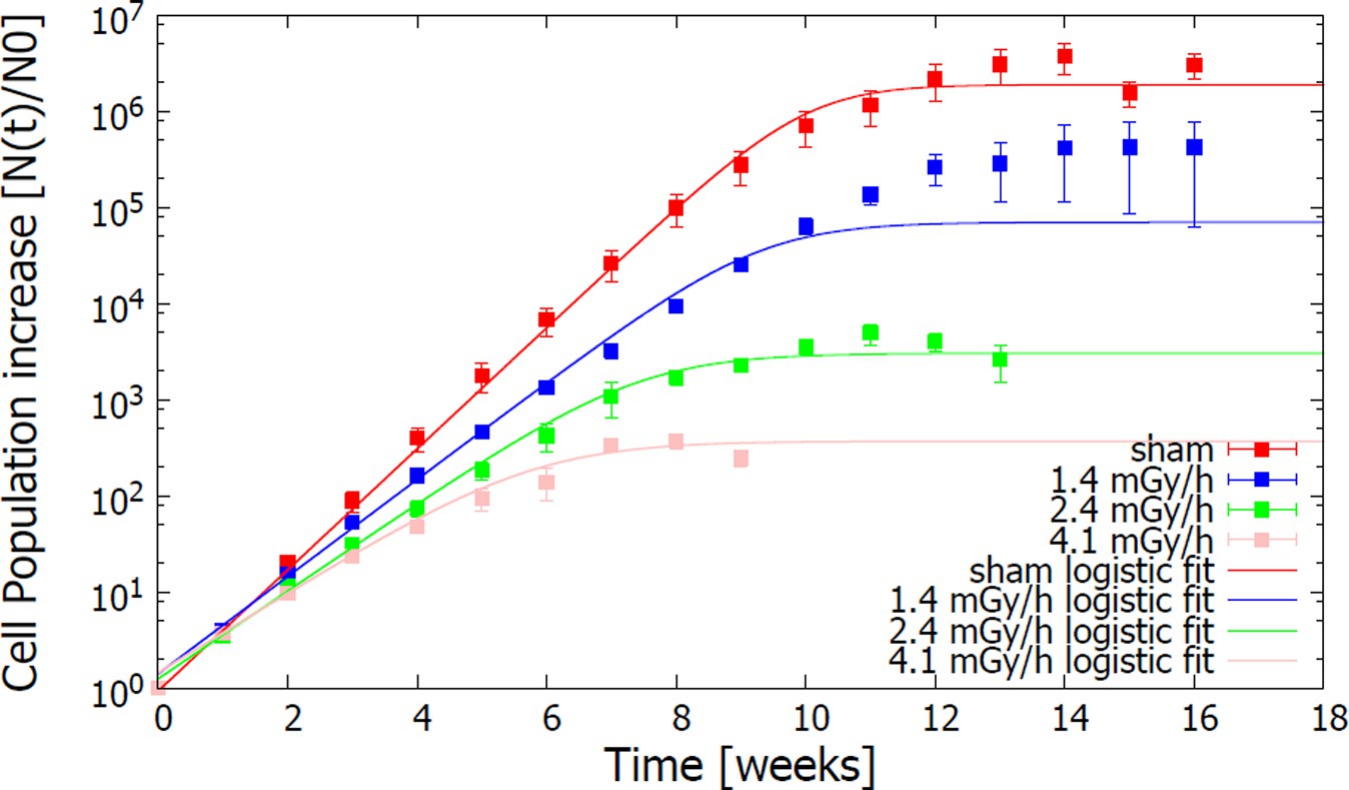
Effects of chronic ionizing radiation exposures on the growth rate of HUVECs. Experimental points are time-series data of the mean cell population increase ±SEM (n = 3) (Yentrapalli, Azimzadeh, Sriharshan, et al. 2013; Yentrapalli, Azimzadeh, Barjaktarovic, et al. 2013). Continuous lines represent the best-fit curves of each dataset, obtained with Eq (3).

The ratio ***N(t)/N***_***0***_ entering the definition of the population doubling (Eq 1) represents the increase in the number of cells as a function of time from the initial seeding. This quantity is plotted in Fig 1 for different exposure conditions: sham irradiated cells (dose rate of 0 mGy/h) and cells exposed to chronic low dose rates (1.4, 2.4 and 4.1 mGy/h) [31,32].

The logistic growth curves of Eq (3) with best-fit parameters ***K***, ***A*** and ***r*** for each exposure condition were also plotted in Fig 1. Logistic growth curves reproduced reasonably well experimental data points for all dose rates, with the exception of the 1.4 mGy/h case. In this latter condition, the logistic curve was not able to reproduce experimental results between week 7 and 12: in this time interval, predictions based on best-fit parameters lead to an overestimation of the weekly increase in the cell population.

To compare the effects of the different conditions, we assessed the variation of the growth rate as a function of the corresponding dose rate. A decreasing trend of the growth rate parameter ***r*** as a function of the dose rate is put in evidence by the values shown in Table 1. The reduction was of approximately 35% for the highest dose rate (4.1 mGy/h) when compared to the sham-irradiated cells.

**Table 1:** Best fit values of the r parameter in Eq (3) as a function of the dose rate.

	Parameter "r"		Parameter "K"		
Dose rate	Value ± SD	% error	Value ± SD	% error	Degrees of freedom
0 mGy/h (sham)	1.450 ± 0.022	1.54%	1.89·10^6^ ± 2.7·10^5^	14.39%	14
1.4 mGy/h	1.164 ± 0.015	1.32%	7.02·10^4^ ± 3.82·10^4^	54.49%	14
2.4 mGy/h	1.052 ± 0.041	3.89%	3.03·10^3^ ± 3.2·10^2^	10.62%	11
4.1 mGy/h	0.960 ± 0.043	4.50%	3.70·10^2^ ± 7.2·10^1^	19.39%	7

Fits to experimental data and statistics were done using Gnuplot Version 5.0 open source software (http://www.gnuplot.info). The "fit" command used in Gnuplot can fit the user-defined functions (Eq (3)) to a set of data points (x, y, y-standard-deviation), using an implementation of the nonlinear least-squares (NLLS) Marquardt-Levenberg algorithm.

### Senescence-associated β-galactosidase staining cells

**Fig 2:**
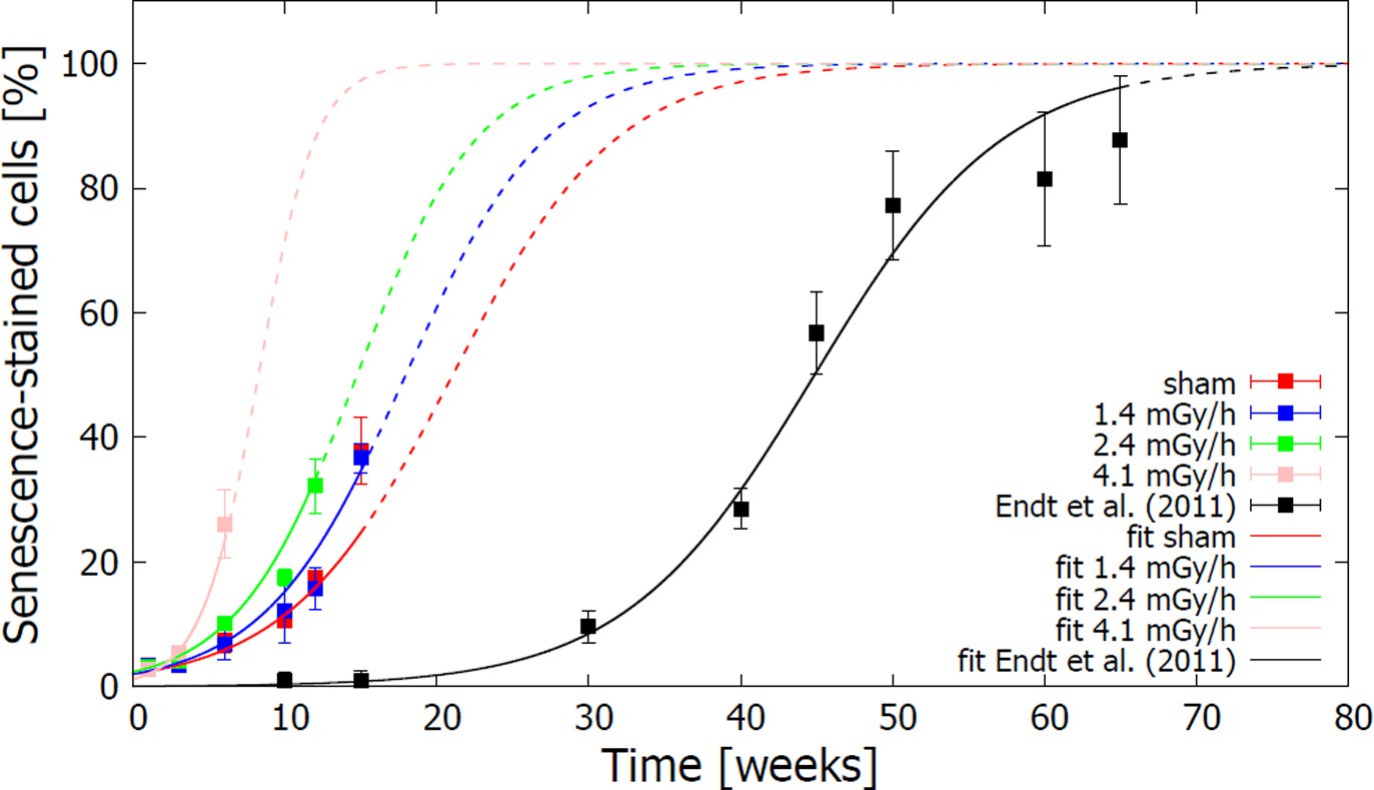
Effects of chronic ionizing radiation exposures on senescence levels of HUVECs. Experimental points are time series data of the mean percentages of SA- β -gal stained cells ± SEM (n = 3) (Yentrapalli, Azimzadeh, Sriharshan, et al. 2013; Yentrapalli, Azimzadeh, Barjaktarovic, et al. 2013). Continuous lines represent the best-fit curves of each dataset, obtained with Eq (5), drawn up to the last experimental time point available. Dashed lines represent the continuation for longer time points of the best-fit curves, to highlight the differences among the obtained graphs.

The percentage ***S(t)*** of cells positive to the senescence marker β-galactosidase at different time points indicated progressing senescence in the cell population and provided a complementary measurement to the population doubling endpoint. In Fig 2, the analysis of the measured ***S(t)*** values showed that the induction of senescence was especially accelerated after exposure of cells to the highest dose rate (4.1 mGy/h), while this effect was less pronounced after exposure to 2.4 mGy/h. Exposure of cells to a chronic dose rate of 1.4 mGy/h did not significantly affect the onset of senescence. Continuous lines in Fig 2 are best-fit curves to ***S(t)*** datasets with Eq (5), via the free parameters ***B*** and ***p*.**

To test whether the assumption of a logistic curve for the percentage of senescent cells as a function of time can be generalized to different conditions and cell populations, we also included in the analysis data by Endt et al. [45] on the replicative senescence of non-irradiated SBL5 primary fibroblasts: their results, together with the corresponding best fit curve with Eq 5, are shown in Fig 2. Logistic curves nicely reproduced experimental data for almost all conditions, and a very good agreement was found on a long time interval (up to about 60 weeks of subcultures) for SBL5 primary fibroblasts. Only at week 15, predictions based on best-fit parameters for sham-irradiated cells lead to an underestimation of the percentage of senescent cells.

The coefficient ***p*** in Eq (5) represents the slope of the growth component of the number of senescent cells as a function of time. When looking at best fit values of ***p*** as a function of the corresponding dose rates, a clear dose-rate dependence was observed (Table 2): the senescence rate increased as a function of the dose rate.

**Table 2:** Best fit values of the p parameter in Eq (5) as a function of the dose rate.

	Parameter "p"		
Dose rate	Value ± SD	% error	Degrees of freedom
0 mGy/h (sham)	0.184 ± 0.027	14.9%	4
1.4 mGy/h	0.215 ± 0.017	7.8%	4
2.4 mGy/h	0.252 ± 0.037	14.6%	3
4.1 mGy/h	0.532 ± 0.102	19.2%	1
Endt et al. data	0.160 ± 0.018	11.3%	6

Fits to experimental data and statistics were done using Gnuplot Version 5.0 open source software (http://www.gnuplot.info). The "fit" command used in Gnuplot can fit the user-defined functions (Eq (5)) to a set of data points (x, y, y-standard-deviation), using an implementation of the nonlinear least-squares (NLLS) Marquardt-Levenberg algorithm.

### Vascular network formation

**Fig 3:**
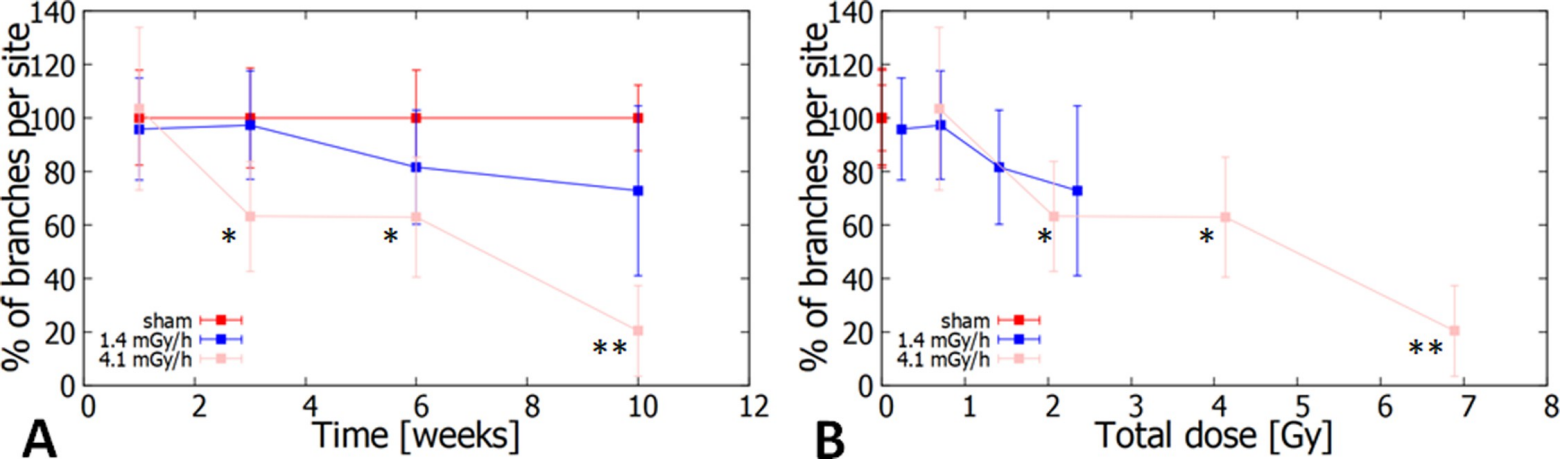
Effects of chronic ionizing radiation exposures on the functionality of HUVECs. Experimental points are percentages of branches per site (average over 10 sites), as a measure of cell capacity to form vascular networks: A) data are plotted as a function of the exposure time; B) data are plotted as a function of the total cumulative dose received. All data are presented as mean ± SD, with n = 3.*p-value<0.05 and **p-value<0.01 versus untreated HUVECs at the same time (Ebrahimian et al. 2015).

The capability of HUVECs to form vascular networks was measured on cells exposed to chronic radiation and corresponding sham-irradiated controls at different time points (up to 10 weeks). Data representing the percentage of branches per site as a function of time, taken from Ebrahimian et al. [39], were compared in Fig 3A: as already highlighted in the publication, a clear loss of cell functions were observed after three weeks of exposure at the highest dose rate (4.1 mGy/h), however such effect was not significantly different for the lowest dose rate (1.4 mGy/h). When analysing the effects as a function of the "total cumulative dose" (i.e. exposure time multiplied by the dose rate, see Materials and Methods), an interesting observation can be done: no difference could be observed between exposure conditions for the same dose ranges, with the 4.1 mGy/h and the 1.4 mGy/h datasets overlapping (Fig 3B). Particular attention should be given to the possible presence of a dose-threshold for the vascular network formation assay: up to about 0.7 Gy, no statistically significant reduction in the HUVEC capacity to form vascular networks can be observed, while a tendency to a cell function loss was observed at higher doses.

### Proteomics and transcriptomics datasets results

Comparison of the proteomic and transcriptomic profiles included the search for both dose-rate- and total dose-dependent effects at fixed time points and at the same cumulative dose, respectively.

The comparisons at fixed time points of merged transcriptomics and proteomics datasets confirmed the results already obtained by Yentrapalli et al. [35,36] and by Rombouts et al. [37]. In particular, results of the transcriptomic and proteomic datasets for the exposure to 4.1 mGy/h for 6 weeks highlighted the onset of oxidative stress as well as of pathways involved in the delicate equilibrium among proliferation, inflammation, senescence and death (as suggested by the perturbation of the mTOR, FoxO, p53 and several other signalling pathways). Among these pathways, the perturbation of Insulin-like Growth Factor (IGF) transport and uptake by Insulin-like Growth Factor Binding Proteins (IGFBPs) confirms the findings by Rombouts et al [37]. Concerning the lowest dose-rate, the transcriptomic data analysis, available up to 6 weeks of exposure, highlighted a milder deregulation of FoxO and mTOR signalling pathways and few metabolism-related pathways (e.g. Drug metabolism cytochrome P450, Retinol and Phosphatidylinositol metabolism).

**Fig 4:**
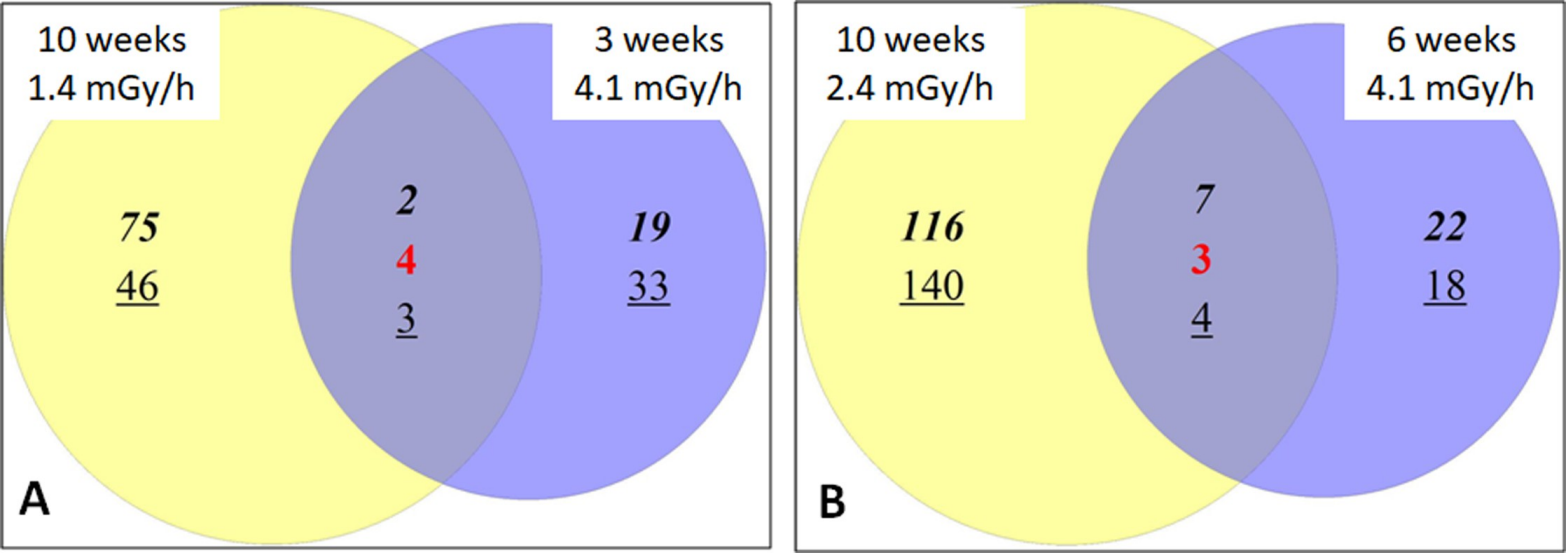
Numbers of deregulated proteins at the different time points and exposure dose rates compared to sham irradiated controls at the same time point. A) Shows the overlapping proteins after 1.4 mGy/h for 10 weeks and after 4.1 mGy/h for 3 weeks (total dose of 2.35 and 2.07 Gy, respectively). B) Shows the overlapping proteins after 2.4 mGy/h for 10 weeks and 4.1 mGy/h for 6 weeks (total dose of 4.0 and 4.1 Gy, respectively). Bold numbers: Up-regulated; Red numbers: Contra-regulated in the two conditions; Underlined numbers: Down-regulated.

The proteomics analysis performed at different time points allowed to perform a comparison of protein expression values in cells exposed to total cumulative doses of 2 and 4 Gy, delivered with the dose rates of 1.4, 2.4 and 4.1 mGy/hs. Comparing the proteomics analysis performed at 10 week exposure at 1.4 mGy/h (2.35 Gy) to that at 3 week exposure at 4.1 mGy/h (2.07 Gy), a great difference regarding the number of deregulated proteins with respect to their respective controls was observed (Fig 4A). At the lowest dose rate (1.4 mGy/h) the number of deregulated proteins was 130 while only 61 proteins were found deregulated at 4.1 mGy/h. Only 9 proteins were in common between the two conditions, among which 2 were up-regulated (ACTN1 and CAPN2), 3 were down-regulated (IDH2, PPIB and PDIA4), and 4 were found contra-regulated in the two conditions (AHNAK, ITGB1, MCAM and PRCP).

Deregulated proteins obtained from the proteomics analysis were analyzed for the associated pathways using the Reactome FI tool in Cytoscape. The analysis highlighted 72 and 90 altered pathways for cells exposed to 1.4 mGy/h and to 4.1 mGy/h, respectively. Out of these, only 45 pathways were found in both proteomic profiles, while 72 pathways were uniquely found in only one of the two conditions.

The effect of a total cumulative dose of approximately 4 Gy was studied comparing results for a 10 week exposure at 2.4 mGy/h (4.0 Gy) to a 6 week exposure at 4.1 mGy/h (4.1 Gy). The overall picture is similar to that obtained for a total cumulative dose of 2 Gy (Fig 4B). The number of deregulated proteins following exposure to 2.4 mGy/h was 270, while for the highest dose rate (4.1 mGy/h) only 54 proteins were affected significantly. Among these, only 14 proteins were in common between the two conditions and, in particular, 7 were found up-regulated (ACTN1, CD44, HSP90AA1, PLBD2, RAB7A, TPR and VCL), 4 down-regulated (HIST1H1E, PPIB, PDIA4 and SERPINH1) and 3 contra-regulated (RCN1, RNH1 and TAGLN2).

For cells exposed to 2.4 mGy/h, 83 pathways were found to be changed, while 116 were altered in the cells exposed to 4.1 mGy/h. Among these pathways, only 40 were found in both conditions and 116 uniquely in one of the two exposure scenarios. Notably, in both conditions pathways connected to senescence were identified, as summarized in Table 3 (from full lists of pathways available in S2 Table). The pathways "Senescence-Associated Secretory Phenotype (SASP)" and "Oncogene Induced Senescence" were found at both dose rates whilst the "DNA Damage/Telomere Stress Induced Senescence" and "Oxidative Stress induced Senescence" were found only in the cells exposed to the higher dose rate for a shorter time.

**Table 3:** List of senescence-related pathways identified for the highest total cumulative dose comparison of the proteomics profiles of 4.1 mGy/h for 6 weeks (right) and 2.4 mGy/h for 10 weeks (left).

	2.4 mGy/h 10 weeks (4.0 Gy)					4.1 mGy/h 6 weeks (4.1 Gy)				
Pathways	Number of protein in gene set	Protein from network	P-value	FDR	Nodes	Number of protein in gene set	Protein from network	P-value	FDR	Nodes
Senescence-Associated Secretory Phenotype (SASP)(R)	50	7	0.0005	7.97E-03	MAPK1, RPS27A, RELA, UBA52, FOS, CDK2, UBC	50	4	0.0004	3.34E-03	NFKB1, MAPK1, UBA52, HIST1H4A
Oncogene Induced Senescence(R)	30	6	0.0002	3.75E-03	MAPK1, ETS1, RPS27A, UBA52, E2F1, UBC	30	3	0.0011	7.32E-03	TP53, MAPK1, UBA52
DNA Damage/Telomere Stress Induced Senescence(R)						26	3	0.0007	5.73E-03	TP53, HMGA1, HIST1H4A
Oxidative Stress Induced Senescence(R)						64	4	0.0009	6.53E-03	TP53, MAPK1, UBA52, HIST1H4A

Another interesting finding obtained from the isodose comparisons is the perturbation of cell cycle related pathways in both dose rates exposures (1.4 and 4.1 mGy/h), when the cumulative dose is approximately 2 Gy, while this similarity is missing when comparing the dose rates (2.4 and 4.1 mGy/h) at about 4 Gy of total dose. In the latter case, the highest dose rate show almost no perturbation to cell cycle related pathways (see S2 Table for details).

## Discussion

The goal of this study was the evaluation of the response of HUVECs to chronic low-dose-rate γ-irradiations and the effects of cumulative doses due to protracted exposures. Results presented in this work provide an insight into mechanisms leading to premature cellular senescence induced by the chronic exposure. To this aim, we collected and reanalysed data on different endpoints, obtained in the framework of a specific subtask of the European project EURATOM FP7 NoE "DoReMi" and previously published separately, to provide additional information and move a step forward towards a complete picture of radiation-induced effects in endothelial cells.

Endpoints analysed in this work are: the proliferation capacity; the senescent status; the capability of forming vascular networks; and both transcriptome and proteome profiles.

Cellular senescence is a phenomenon that has been observed both in *in vitro* and in *in vivo* models. It is typically found in cells belonging to renewable tissues or those showing prolonged inflammatory status. Senescent cells reside in a cell cycle arrested state protecting them from the accumulation of damage and the potential transmission of malignant mutations to their progeny [46,47]. The first evidence of senescence was showed by Hayflick and Moorhead as a loss of proliferative capability, thereby called "replicative senescence" [48], and due to telomere shortening. Cellular senescence can occur also independently of telomere shortening, being induced by a wide range of possible stressors, among which oxidative stress or severe DNA damages are common. This "stress-induced senescence" can be triggered by either physical or chemical insults, such as exposures or chemotherapy [49].

Starting from the phenotypic aspects, such as the proliferation rate and the senescence status, a phenomenological interpretation of the collected experimental data has been introduced. We proposed a simplified mathematical model, looking only at the "collective" phenomena of cell proliferation and senescence and not at the molecular mechanisms underpinning such macroscopic effects. The logistic growth curve is proposed as a mathematical description of the temporal growth of the cell population, justified by physical (space) and physiological (nutrients) constraints of primary cell cultures. This model nicely reproduced the increase of HUVEC population over approximately 20 weeks. The logistic curve failed to reproduce data only for the intermediate time points of the lowest dose rate (1.4 mGy/h), i.e. between week 7 and 12. A possible explanation is that the model is more suitable to predict the behaviour of cells experiencing permanent growth reduction rather than predicting milder effects on cell growth. This could be also related to the higher heterogeneity observed in the response for the low dose rate 1.4 mGy/h: indeed, cells exposed to the lowest dose rate, although initially growing slower than sham-irradiated cells, seem to reach a population size close to that of the control condition after 16 weeks (see Fig 1). Interestingly, the growth rate parameter ***r*** obtained from the fit to experimental data show a decreasing trend as a function of the dose rate at which cells were exposed.

Data obtained by the senescence-associated β-galactosidase staining provided a possible explanation for the decreased growth rate following radiation exposure. The same mathematical model (logistic curve) for the growth of the cell population was applied to the senescence data to better evaluate the potential dose-rate dependence. Fit curves demonstrate the capability of the model to reproduce experimental data. The parameter ***p*** was extracted, giving the slope of the exponential growth in number of senescent cells as a function of time. As expected, best fit values of this parameter increase as a function of the dose rate.

Given the limited number of data points, simple linear dependences with opposite slopes were tested for the parameters ***r*** in the growth curve and ***p*** in the increase of senescence as a function of dose rate. In both cases however, a linear-quadratic dependence cannot be ruled out, and would require further investigation to be confirmed.

The logistic model for the percentage of senescent cells was also tested on a completely different dataset performed on SBL5 primary fibroblasts from Endt et al. [45], demonstrating its validity on different cell lines. The difference in replicative senescence of different cells (HUVEC and SBL5) constituted *per se* interesting information. However, as cells in Endt et al. data were not exposed to radiation, it was not possible to expand the conclusion on radiation-induced premature senescence.

The results obtained regarding the growth rate and senescence status were followed by the study of the capability of HUVECs to produce vascular networks. The original work was performed by Ebrahimian et al. [39], highlighting the loss of HUVEC angiogenic functions at the highest dose rate (4.1 mGy/h), while almost not statistically significant detrimental effects could be seen at the lowest one (1.4 mGy/h). Complementary analysis of the inflammatory cytokines released by the HUVECs [39] highlighted the strong relationship between these processes, as also previously confirmed [50]. A comparison of angiogenic functions at different time points and a comparison of angiogenic functions following different total doses provided an insight into the possible dose rate and dose dependencies. Indeed, the loss of capability in forming vascular networks seemed to be dependent on the dose rate, as seen at weeks 3, 6 and 10 for the highest dose rate 4.1 mGy/h, as well as on the total dose, as seen for the total cumulative radiation doses 0.69, 2.1, 4.1 and 6.9 Gy calculated from the dose rate 4.1 mGy/h at 1, 3, 6 and 10 weeks, as previously confirmed [39], However, comparison of the total dose is limited to 2.3 Gy for 1.4 mGy/h at the longest exposure time (10 weeks) which restricts the possibility of observing differences between dose rates at higher total doses. A further interesting aspect arising from this analysis is the possible presence of a dose-threshold of 0.7 Gy, independent of the dose rate, below which no effects on the angiogenic functions have be seen. Of note, the International Commission on Radiological Protection (ICRP) notes an acute dose threshold of approximately 0.5 Gy for circulatory disease (both cardiovascular and cerebrovascular diseases) [51]. An almost total loss of angiogenic function (20% of the control condition) is seen at approximately 7 Gy. These results on chronically exposed HUVEC can be compared to acutely exposed HUVEC at similar doses, showing a significant loss of vascular network formation for doses higher than 4 Gy, as described in the work by Ahmad et al. [52].

In contrast to the angiogenic properties, the proteomics study showed distinct alterations depending on both the dose rate and the total dose. The data emphasized that different dose rates induced distinct molecular mechanisms in the cells during the onset of senescence. The highest dose rate affected several senescence-related biological pathways, among which cell-to-cell communication, cellular adhesion, and inflammation. Instead, the lowest and moderate dose rates showed a milder effect [35]. Nonetheless, the dose rate of 2.4 mGy/h was able to induce premature senescence, expression of cell cycle related inhibitors such as cyclin-dependent kinase inhibitor p21, as well as the inhibition of the PI3K/Akt/mTOR pathway. Inhibition of PI3K pathway and reduced NO has been reported after 500 mGy IR acutely exposed human coronary artery endothelial cells [53]. The analysis of cardiac endothelial cells isolated form murine hearts locally exposed to high dose irradiation also showed similar proteome changes. The main affected pathway was INS (IGF)/PI3K/AKT/eNOS signalling cascade. Inhibition of PI3K pathway results in reduced eNOS phosphorylation and reduced NO production, that is known as hallmark for endothelial dysfunction [54].

In line with the proteomic data, the transcriptomic analysis by Rombouts et al. [37] using the same exposed HUVECs highlighted both an inflammation-related profile (starting from 3 weeks of exposure with the dose rate of 4.1 mGy/h) and a significant increase of IGFBP5, a factor known for reducing the bioavailability of IGF, and subsequently inhibition of PI3K/AKT/mTOR.

Additional analysis comparing the proteome alteration at fixed total cumulative doses, but delivered using different dose rate (and hence exposure times), evidenced a strong difference between the two dose conditions (*i*.*e*. approximately 2 Gy and 4 Gy). At 2 Gy, although the numbers of deregulated proteins at different dose rates were different, several pathways were found to be in common. None of the altered pathways, however, was linked to the senescence state, as expected from the results on the β-gal stained cells. By reaching the total dose of 4 Gy the damage in HUVECs seemed to be more severe as previously reported in other types of endothelial cells [55–58]. Several pathways connected to senescence were found at 4 Gy. Interestingly, although the delivered doses were almost identical, cells that received the dose with the highest dose rate were also showing a more significant senescent status based on pathway analysis when compared to cells exposed to the intermediate dose rate (2.4 mGy/h).

The potential of the approach used for the analysis could have been enhanced by the inclusion of more time points, especially for the low-dose-rate experiments, to observe differences between dose rates at higher total doses, and the inclusion of more endpoints, such as oxidative stress, cell death and DNA damage, to have a more complete picture of radiation-induced effects in endothelial cells. In addition, it would be interesting to include more -omics data, such as metabolomics, which can provide a ‘common denominator’ to the analysis of multi-omics experiments due to its “closeness” to cellular or tissue phenotypes. These considerations could set the basis for future experiments.

## Conclusion

In this work we presented an integrated analysis of previously published data for the evaluation of the response of HUVECs to chronic low-dose-rate γ-irradiations. The proposed analysis represents an advance over information on different cellular endpoints given separately, also putting in evidence a strong dose-dependence in the measured endpoints, in some cases with a clear dose threshold (as for the loss of vascular network formation capability). Nonetheless, the scenario is much more complicated and emphasized that the total cumulative dose is certainly not the only factor governing cell response to irradiation: different dose rates affect HUVECs in a distinct manner, not only by accelerating the onset of the senescent status and shortening the life span of the endothelial cells, but also by modifying the sustaining mechanisms leading to alterations in the proliferative status or the vascular network formation. Though data collected for this work present some challenges for the optimal integration, this collaborative effort demonstrates the importance of a combined experimental and modelling approaches to integrate different but complementary endpoints. The findings presented here offer a proof of the advantages of a systems biology approach to achieve a more complete understanding of the mechanisms underlying the response to radiation exposure.

## Supporting information

S1 Table Overview of the available dose rates and time points of the transcriptomic and proteomic data. (DOCX)

S2 Table Pathway analysis from the proteomic dataset using the Reactome FI tool in Cytoscape. (XLSX)
