# Supplementary material for: A systems radiation biology approach to unravel the role of chronic low-dose-rate gamma-irradiation in inducing premature senescence in endothelial cells
Source: PLoS One. 2022 Mar 14;17(3):e0265281. doi: 10.1371/journal.pone.0265281 (PMC8920222; doi:10.1371/journal.pone.0265281)
Supplement: Supplementary file 1 [file pone.0265281.s001.docx]

	1.4 mGy/h				2.4 mGy/h				4.1 mGy/h		
	1 week	3 weeks	6 weeks	10 weeks	1 week	3 weeks	6 weeks	10 weeks	1 week	3 weeks	6 weeks
**Transcriptome**	x	x	x						x	x	x
**Proteome**				x				x	x	x	x
